# Ranking de los programas de inmunización en América Latina, 2019

**DOI:** 10.26633/RPSP.2022.204

**Published:** 2022-12-15

**Authors:** María Fernanda Rombini, Romina Paola Mauas, Analía Urueña

**Affiliations:** 1 Centro de Estudios para la Prevención y Control de Enfermedades Transmisibles Universidad Isalud Ciudad Autónoma de Buenos Aires Argentina Centro de Estudios para la Prevención y Control de Enfermedades Transmisibles, Universidad Isalud, Ciudad Autónoma de Buenos Aires, Argentina.

**Keywords:** Observatorios de salud, programas de inmunización, coberturas de vacunación, vacunación, América Latina, Immunization programs, vaccination coverage, vacination, Latin America, Programas de imunização, cobertura vacinal, vacinação, América Latina

## Abstract

**Objetivo.:**

El objetivo del presente trabajo ha sido construir un *ranking* de los programas nacionales de inmunizaciones (PNI) de América Latina que compare las diversas realidades, identifique los desafíos y metas no alcanzadas, y estimule a los países a la búsqueda de estrategias superadoras.

**Métodos.:**

Se seleccionaron 10 países con los calendarios nacionales de vacunación (CNV) más innovadores. Se utilizó la información publicada en sitios oficiales de los ministerios de salud, la Organización Mundial de la Salud (OMS), la Organización Panamericana de la Salud (OPS), el Fondo de las Naciones Unidas para la Infancia (UNICEF, por su sigla en inglés) y entrevistas a referentes de cada país. Se construyó un *ranking* con base en los dominios vinculados al CNV 2019 en diferentes etapas de la vida, vacunación antigripal, en situaciones especiales, coberturas de vacunación (CV) de 2018 y aspectos programáticos.

**Resultados.:**

El *ranking* general lo lideran Chile y Panamá, con la vacunación del primer y segundo año de vida. Les siguen Argentina, Uruguay y Costa Rica, que se destacan en vacunación de otros grupos, antigripal y aspectos programáticos. Brasil, Colombia y México muestran CNV más atrasados, brechas programáticas y CV más bajas. Por último, Paraguay y Perú presentan carencias similares y mayores vacíos de información. Sin embargo, al analizar los dominios de manera individual, el *ranking* se modifica y no se repite un mismo patrón.

**Conclusiones.:**

Este es el primer ranking de los PNI de América Latina en el que se destacan las fortalezas y debilidades de cada país. La periodicidad de este ejercicio será clave para comparar la evolución y el posicionamiento de estos programas en el tiempo.

## Abstract (en)

**Objective.:**

To establish a ranking of national immunization programs in Latin America that compares the various realities, identifies challenges and unachieved goals, and encourages countries to search for strategies to overcome the obstacles they face.

**Methods.:**

Ten countries with the most innovative national vaccination schedules (NVCs) were selected. Information published on official websites of ministries of health, the World Health Organization (WHO), the Pan American Health Organization (PAHO), and the United Nations Children's Fund (UNICEF) were used, as well as interviews with experts from each country. A ranking was constructed, based on domains linked to the 2019 NVC (vaccinations given at different life stages, influenza vaccination, and vaccination in special situations); vaccination coverage in 2018; and programmatic aspects.

**Results.:**

The general ranking is led by Chile and Panama, due to vaccination in the first and second year of life. They are followed by Argentina, Uruguay, and Costa Rica, which stand out for vaccination of other groups, influenza vaccination, and programmatic aspects. Brazil, Colombia, and Mexico have more delays in their NVCs, as well as programmatic gaps and lower vaccination coverages. Finally, Paraguay and Peru have similar deficiencies and bigger information gaps. However, when the domains are analyzed individually, the ranking changes and the same pattern is not repeated.

**Conclusions.:**

This is the first ranking of national immunization programs in Latin America that highlights strengths and weaknesses in each country. The periodicity of this exercise will be key when comparing the evolution and positioning of these programs over time.

## RESUMO

**Objetivo.:**

O objetivo deste trabalho foi construir um ranking dos programas nacionais de imunização (PNI) da América Latina que comparasse as distintas realidades, identificasse os desafios e as metas não alcançadas e estimulasse os países a buscarem melhores estratégias.

**Métodos.:**

Foram selecionados 10 países com os Calendários Nacionais de Vacinação (CNV) mais inovadores. Foram utilizadas informações publicadas nos sites oficiais dos ministérios da Saúde, da Organização Mundial da Saúde (OMS), da Organização Pan-Americana da Saúde (OPAS) e do Fundo das Nações Unidas para a Infância (UNICEF), e entrevistas com lideranças de cada país. Foi construído um ranking com base nos domínios vinculados aos CNV de 2019 em diferentes fases da vida, vacinação contra influenza, situações especiais, cobertura vacinal (CV) em 2018 e aspectos programáticos.

**Resultados.:**

O ranking geral é liderado pelo Chile e pelo Panamá, com a vacinação no primeiro e no segundo ano de vida. Seguem-se Argentina, Uruguai e Costa Rica, que se destacam na vacinação de outros grupos, na vacinação contra a influenza e em aspectos programáticos. Brasil, Colômbia e México apresentam CNV mais atrasados, lacunas programáticas e CV mais baixas. Por último, Paraguai e Peru apresentam deficiências semelhantes e mais lacunas de informação. No entanto, ao se analisar os domínios individualmente, o ranking se modifica e não se repete um mesmo padrão.

**Conclusões.:**

Este é o primeiro ranking dos PNI da América Latina em que são destacados os pontos fortes e fracos de cada país. A periodicidade deste exercício será fundamental para comparar a evolução e o posicionamento destes programas ao longo do tempo.

El programa ampliado de inmunización (PAI) liderado por la Organización Panamericana de la Salud (OPS) ha sido emblemático para la Región de las Américas, dado que ha logrado erradicar, eliminar y controlar diversas enfermedades prevenibles por vacunación. La Región ha logrado uno de los niveles más altos de coberturas de vacunación en el mundo; sin embargo, aún existen desigualdades entre países y dentro de un mismo país (1, 2). Los principales objetivos de los programas nacionales de inmunizaciones (PNI) de cada país son evitar el estancamiento del índice de cobertura, aumentarlo de manera progresiva y garantizar su homogeneidad, la introducción sostenible de vacunas nuevas y combinaciones también nuevas, así como la ampliación de los calendarios de la infancia a otras etapas de la vida. Algunos de los problemas que enfrentan los PNI es disponer de información adecuada sobre la población objetivo con el fin de extender la vacunación a todas las personas, promover la vacunación oportuna, mejorar las coberturas e informarlas en forma precisa y en tiempo real. Debido a esto, se ha promovido sistematizar el análisis y el uso de los datos de inmunización, evaluar su calidad y crear registros nominales de vacunación informatizados (3). Los PNI, además, han promovido la conformación de comités asesores sobre prácticas de inmunización (CAPI) que aportan transparencia y son de vital importancia para la toma de decisiones avaladas por equipos técnicos independientes y con gran experiencia en cada país (4, 5).

Los rankings en salud tienen como misión ayudar a las comunidades a comprender cuáles son los factores que influyen en la salud de las poblaciones, detectar los problemas e identificar las oportunidades para mejorar estos resultados. Algunos clasifican y comparan índices hospitalarios, otros miden indicadores de salud a nivel jurisdiccional dentro de un mismo país o estado, y otros monitorean y comparan sistemas de salud a nivel país (6-9).

En el presente estudio se construyó un *ranking* de los PNI de la Región de las Américas, a través de la asignación de puntajes y del análisis transparente y metódico de variables relacionadas con las vacunas incluidas en el calendario en diferentes etapas de la vida y en situaciones especiales, la vacunación antigripal, las coberturas de vacunación y los aspectos programáticos. El propósito de este *ranking* es evaluar y comparar las diversas realidades de los países, detectar los problemas y las metas aún no alcanzadas, y estimular a los países a mejorar los aspectos pendientes y aplicar estrategias que funcionan.

## MATERIALES Y MÉTODOS

Se realizó un estudio observacional descriptivo cualicuantitativo. Se eligieron 10 países (Argentina, Brasil, Chile, Colombia, Costa Rica, México, Panamá, Paraguay, Perú y Uruguay) con los calendarios nacionales de vacunación (CNV) más innovadores, producto de las incorporaciones de vacunas como las vacunas contra la hepatitis A (VHA), la varicela (VZV), la vacuna antimeningococo conjugada (MenC o MenACWY), el esquema primario de cuádruple (difteria, tétanos, ton convulsa y *Haemophilus influenzae* de tipo B, DTPHib) con vacuna acelular o combinada con vacuna inactivada contra la polio (pentavalente acelular o hexavalente), o la vacuna contra el virus del papiloma humano en los varones en los últimos 5 años.

### Fuentes de información y técnica de recopilación de datos

Entre febrero y octubre de 2020 se realizó una búsqueda de información pública y de acceso libre en sitios web de los ministerios de salud de los países seleccionados, sitios oficiales de la Organización Mundial de la Salud (OMS), la OPS y el Fondo de las Naciones unidas para la Infancia (UNICEF, por su sigla en inglés). Se priorizó la información ministerial. Además, se realizaron entrevistas a directores de PNI o referentes en inmunización de sociedades científicas de cada país para relevar aspectos cualitativos vinculados, en particular, con el sistema nominal de registro de vacunación (SNRV), la notificación de eventos adversos supuestamente atribuidos a la vacunación o la inmunización (ESAVI) y el funcionamiento de los CAPI. Para ello no se utilizó un instrumento específico, sino que se dirigieron las preguntas en función de la información faltante o discordante del instrumento general (véanse las tablas 1 y 2 en el Material suplementario).

Se analizó el CNV del año 2019, las coberturas de vacunación nacionales y jurisdiccionales del 2018 (al momento de la investigación no se habían cerrado los datos administrativos de coberturas del 2019), guías y manuales de inmunización para la población general, para las situaciones especiales y para la vacunación antigripal de cada país. Se incluyeron todas las vacunas y combinaciones de vacunas que se encuentran en el programa del Fondo Rotatorio de la OPS y otras vacunas que adquieren los países por otras vías (6). La falta de publicación del dato de cobertura para una vacuna determinada se consideró como 0%.

De los aspectos programáticos, se revisaron las leyes sobre vacunas y sus actualizaciones, la existencia de CAPI y difusión de actas públicas, el abastecimiento de vacunas, el acceso a informes sobre ESAVI en papel y en línea (no se incluyó la existencia de un comité de ESAVI en funciones), el control de enfermedades prevenibles por vacunación, la existencia y el alcance de un SRNV, el presupuesto y el gasto teórico en vacunas. El gasto se estimó por persona y por producto bruto interno (PBI) del país, con base en el costo de las vacunas informado por el Fondo rotatorio de la OPS, la población objetivo a vacunar con cada producto y el PBI del 2019 según datos del Banco Mundial (10, 11).

### Identificación de dominios, variables y categorías

Se diseñó una base de datos común para cada país. Se definieron seis dominios: vacunas en el primer año de vida; desde el segundo año de vida hasta el ingreso escolar; en adolescentes, personas embarazadas, personas adultas y personas mayores; vacunación antigripal; vacunación en situaciones especiales; y aspectos programáticos. Las variables en cada dominio se refieren a la presencia en calendario de una vacuna definida, a una determinada indicación de esa vacuna o a procesos o indicadores (solo en aspectos programáticos) (tabla 1 del Material suplementario).

A su vez, para algunas variables se definieron categorías vinculadas al uso de vacunas combinadas, acelulares y de mayor cobertura microbiológica, índices de cobertura de 2018, el alcance del SNRV y la medición del gasto en vacunas (tabla 2 del Material suplementario).

**CUADRO 1.** Dominios y puntaje por dominio y total

Conjunto de variables agregadas y sus categorías	Número de variables a las que se le asignan puntaje	Puntaje máximo con las categorías
Vacunas en el primer año de vida	28	125
Vacunas desde el segundo año de vida hasta el ingreso escolar	15	77
Vacunas en adolescentes desde los 9 años, personas embarazadas, personas adultas y personas mayores	12	59
Vacuna para la gripe (grupos prioritarios)	22	42
Situaciones especiales	30	30
Aspectos programáticos e indicadores	41	64
Total	148	397

*Fuente:* elaboración propia.

**CUADRO 2.** Información de acceso público sobre los 10 países estudiados

	Argentina	Brasil	Chile	Colombia	Costa Rica	México	Panamá	Paraguay	Perú	Uruguay
Página web del ministerio de salud	Sí	Sí	Sí	Sí	Sí	Sí	Sí	Sí	Sí	Sí
Calendario del 2019	Sí	Sí	Sí	Sí	Sí	Sí	Sí	Sí	Sí	Sí
Coberturas del 2018 publicadas por el país	Sí	Sí	Sí	Sí	No	No	Sí	No	Sí	No
Normas y manuales de vacunación (año de publicación)	2013	2019	2018	2020	2013	2017	2012	2017	2018	2018
Manual para situaciones especiales	Sí	Sí	Sí	No	No	Sí	Sí	Sí	No	Sí
Comité asesor en prácticas de inmunización (CAPI)	Sí	Sí	Sí	Sí	Sí	Sí	Sí	Sí	Sí	Sí
Actas públicas del CAPI	Sí	Sí	Sí	No	Sí	Sí	Sí	Sí	Sí	Sí
Notificación de ESAVI en línea	Sí	No	Sí	Sí	Sí	Sí	Sí	Sí	Sí	Sí

*Fuente:* elaboración propia.

ESAVI: eventos adversos supuestamente atribuibles a la vacunación o inmunización.

### Puntaje

Para cada variable y categoría se adjudicó un 0 si no se disponía y un 1 si se disponía, para obtener un puntaje final por dominio y otro total que incluye los seis dominios evaluados. El máximo puntaje potencial, con base en el supuesto de que un país disponga de 100% de las variables y sus categorías sería de 397 puntos (cuadro 1).

## RESULTADOS

### Información pública y de libre acceso

Todos los ministerios de salud de los países tienen página web con datos de acceso abierto. Así, se logró acceder a los 10 calendarios de vacunación del 2019 (12-21). Las coberturas de vacunación se obtuvieron mediante el acceso también abierto a los sistemas de registro nacionales (18-21), a los boletines oficiales del país (22, 23) o, en su defecto, a fuentes de datos de organismos internacionales como la OPS y la OMS (1,2).

En todos los países seleccionados se hallaron recomendaciones, normas, lineamientos técnicos y manuales de vacunación y CAPI (2, 28-37). Además, Argentina, Brasil, Chile, México, Panamá, Paraguay y Uruguay publicaron recomendaciones para situaciones especiales (29, 30 ,34-36, 38, 39) (cuadro 2).

### Calendarios de vacunación

Todos los países indican la vacuna BCG al nacer y, excepto Uruguay, vacunan contra la hepatitis B en las primeras horas de vida. Argentina, Brasil, Colombia, Paraguay, Perú y Uruguay utilizan la vacuna pentavalente contra la difteria, el tétanos, la tos convulsa, la enfermedad por *Haemophilus influenzae* de tipo B y la hepatitis B (DTP/Hib/VHB); Chile y Panamá utilizan la vacuna hexavalente (las anteriores más la vacuna contra la poliomielitis; dTpa/Hib/VHB/IPV), y Costa Rica y México usan la pentavalente acelular (PVa) (dTpa/Hib/IPV). La totalidad de los países contemplan la vacunación contra la hepatitis B a los 2, 4 y 6 meses. Para el 2019, solo tres países (Chile, Costa Rica y Uruguay) habían suspendido por completo la aplicación de la vacuna oral contra la poliomielitis (bOPV) durante el primer año de vida. Todos los países inmunizan contra el neumococo con una vacuna conjugada (VCN). Solo Argentina y Brasil tienen en el calendario una vacuna conjugada contra meningococo en el primer año de vida (MenACWY y MenC, respectivamente).

En 2019, durante el segundo año de vida, todos los países indicaron vacuna triple vírica (contra sarampión, paperas y rubéola, SRP), contra la varicela (excepto Chile y México) y un refuerzo de vacuna conjugada contra neumococo. Brasil, además, contempla la vacuna tetravírica (SRP y varicela) a los 15 meses. Todos los países utilizan refuerzo de DTP durante el segundo año de vida. En dicha etapa, Argentina, Brasil y Chile incluyen la vacuna contra el meningococo con refuerzo o primera dosis. Siete de los 10 países seleccionados indican una o dos dosis de vacuna para la hepatitis A. Nueve de los 10 países contemplan el primer refuerzo de vacuna antipoliomielítica en esta etapa y, al 2019, solo Uruguay había dejado de administrar un refuerzo en esta etapa y contaba con un esquema completo de cuatro dosis de vacuna contra la poliomielitis inactivada (IPV).

Todos los países indican la vacuna contra la gripe estacional. Argentina y Colombia la aplican en la población de entre 6 y 24 meses de edad. Paraguay y Perú la extienden hasta los 35 meses, mientras que Brasil, Chile, Costa Rica, México, Panamá, y Uruguay lo hacen hasta los 59 meses.

Seis países indican en sus calendarios vacuna dTpa en adolescentes y tres contemplan vacuna dT; Colombia no la incluyó en esta etapa. Además, en el período estudiado todos los países incorporaron vacuna contra el virus del papiloma humano (VPH) en la adolescencia, algunos países incluso la indican en varones. Solo Argentina y Brasil indican refuerzo de vacuna conjugada contra meningococo en adolescentes. En el caso de los adultos, excepto Chile y Colombia, todos los países seleccionados recomiendan vacunación dT. Los únicos dos países que indican vacunación contra hepatitis B en el adulto son Argentina y Brasil.

**FIGURA 1.**
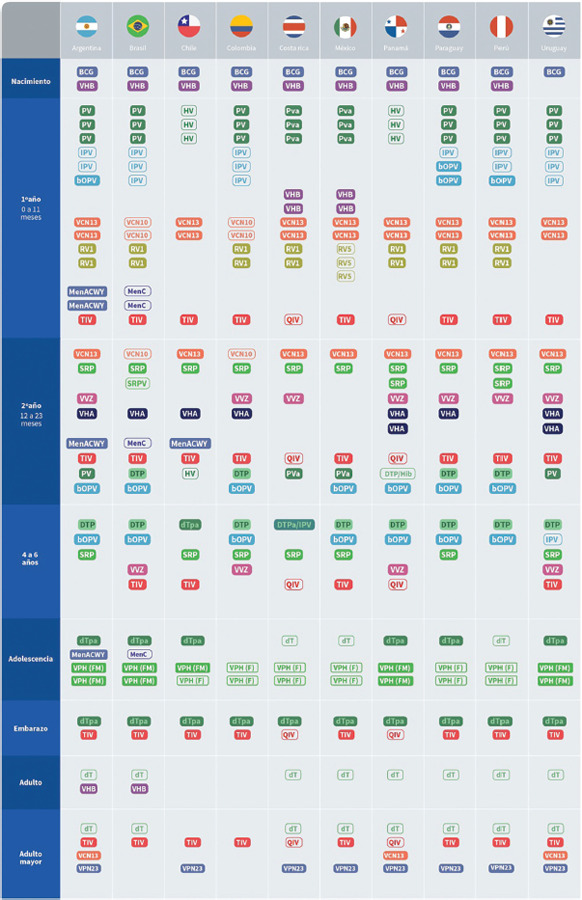
Calendarios de vacunación de algunos países de América Latina (2019)

Todos los países indican vacunación con dTpa y antigripal a personas embarazadas. Además, todos indican vacunación antigripal en personas mayores y ocho contemplan refuerzo de dT. Por último, Argentina, Panamá y Uruguay recomiendan la vacunación contra el neumococo con vacuna conjugada 13 valente (VCN13) y polisacárida (VPN 23) en esquema secuencial. Colombia y Brasil no tienen ninguna de estas en su esquema de rutina y los demás utilizan una u otra (figura 1).

*Fuente:* elaboración propia a partir de la información disponible en los calendarios nacionales de vacunación de los 10 países estudiados.

VHB, vacuna contra la hepatitis B; PV, vacuna pentavalente (DTP/Hib/VHB); Pva, vacuna pentavalente acelular (DTPa/Hib/IPV); HV, vacuna hexavalente (DTPa/Hib/HBV/IPV); IPV, vacuna contra la poliomielitis inactivada; bOPV: vacuna contra la poliomielitis oral bivalente; VCN10, vacuna conjugada contra el neumococo de 10 serotipos; VCN13, vacuna conjugada contra el neumococo de 13 serotipos; RV1: vacuna contra el rotavirus monovalente; RV5, vacuna contra el rotavirus pentavalente; MenACWY, vacuna contra el meningococo conjugada tetravalente (serogrupos ACWY135); MenC: vacuna antimeningococo monovalente (serogrupo C); TIV, vacuna antigripal trivalente (por su sigla en inglés); QIV, vacuna antigripal cuadrivalente (por su sigla en inglés); SRP, vacuna triple viral contra el sarampión, la rubéola y las paperas; SRPV, vacuna cuádruple viral (SRP y varicela); VVZ, vacuna contra la varicela zóster; VHA, vacuna contra la hepatitis A; VPH (F/FM), vacuna contra el virus del papiloma humano VPH para la población femenina y para la femenina y masculina, respectivamente; VPN23, vacuna polisacárida contra el neumococo.

### Coberturas de vacunación

En el 2018, la meta de cobertura >95% para DPT1, DPT3, POL3 y SRP1 se alcanzó en 4, 1, 1 y 5 países, respectivamente. La proporción de municipios con cobertura >90% es baja en la mayoría de los países, y solo en Chile supera el 70% de municipios con una cobertura >90% en las cuatro vacunas estudiadas.

Del total de variables que requirieron del dato de cobertura vacunal, se consideró 0% en 34% de los casos: 20% por falta de incorporación de la vacuna al calendario y 14% por falta de publicación del dato. Esta proporción fue mayor para las vacunas desde el segundo año de vida hasta el ingreso escolar (24% por falta de incorporación de la vacuna al calendario y 13% por falta de publicación del dato) y para las indicadas en adolescentes, personas embarazadas y personas adultas (38 y 32%, respectivamente).

### Ranking de los países por dominios

**FIGURA 2.**
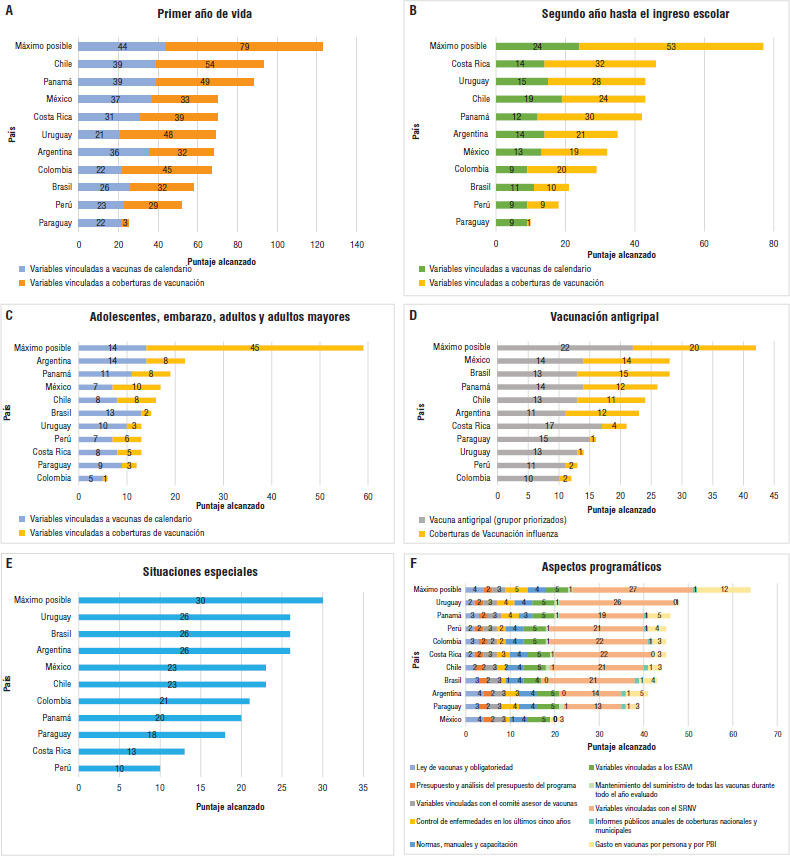
*Ranking* de los programas nacionales de inmunizaciones según los dominios, 2019. A: primer año de vida; B: segundo año de vida hasta el ingreso escolar; C: adolescentes, personas embarazadas, personas adultas y personas mayores; D: vacunación antigripal; E: situaciones especiales; F: aspectos programáticos

En la figura 2 se muestra el orden de los países en el *ranking* según el puntaje alcanzado en cada dominio analizado. Las principales diferencias en los esquemas de calendario tienen que ver con la utilización de la vacuna dTpa, IPV, vacunas combinadas como la tetravírica o la hexavalente, y nuevas vacunas como las vacunas contra la varicela, el meningococo y el virus de la hepatitis A (VHA).

Al considerar el primer año de vida, Chile y Panamá alcanzaron un puntaje mayor tanto en los esquemas de calendario, vinculado al uso de la vacuna hexavalente, como en las coberturas vacunales alcanzadas en esta etapa de la vida. En la vacunación del segundo año de vida y hasta el ingreso escolar nuevamente se destacó Chile en la inclusión de vacunas de calendario relacionado con el uso de la vacuna hexavalente. Sin embargo, Uruguay y Costa Rica alcanzaron un puntaje mayor para este grupo de variables a expensas de mayores coberturas de vacunación. En esta etapa comenzó a evidenciarse una brecha mayor entre el puntaje máximo posible y el real de las coberturas de vacunación, que podría relacionarse con la falta de publicación del dato de coberturas de vacunación contra el meningococo, el VHA y la varicela.

En adolescentes, personas embarazadas y personas mayores, Argentina, Brasil, Panamá y Uruguay mostraron los mayores puntajes asociado al uso de vacuna dTpa, antimeningocócica y contra el VPH en mujeres y varones, vacunación antineumocócica secuencial y vacuna contra el VHB universal. Se observó falta de información en relación con las coberturas de vacunación, principalmente en personas adultas y personas mayores.

México y Brasil alcanzaron el mayor puntaje cuando se combinaron los grupos de riesgo y las coberturas alcanzadas en vacunación antigripal. Sin embargo, en relación con la inclusión al calendario de la vacuna fueron superados por Paraguay, Uruguay, Panamá y Costa Rica, este último vinculado al alcance en pediatría hasta los 6 años y al uso de la vacuna antigripal cuadrivalente (QIV, por su sigla en inglés), que también ha incluido Panamá. Llaman la atención las coberturas bajas y los vacíos de información que presentan Colombia, Costa Rica, Paraguay, Perú y Uruguay en este dominio.

Los primeros lugares en el ranking de vacunación de poblaciones especiales los ocuparon Argentina, Brasil y Uruguay. Esto se debe a la inclusión de vacunas para grupos priorizados de personas inmunocomprometidas o con enfermedades crónicas, como así también en personal de salud, personas viviendo con VIH y hombres que tiene sexo con hombres (HSH).

Por último, en relación con los aspectos programáticos analizados, tanto el país que más se destaca en este dominio (Uruguay) como el que menos puntaje obtuvo (México), tuvieron relación con el mayor o el menor alcance y el nivel de implementación del SNRV. En relación con el mantenimiento del suministro de vacunas en 2019, Argentina, Brasil y México presentaron faltantes de vacunas. Por otra parte, Costa Rica México y Uruguay no publican anualmente sus coberturas de vacunación. Las demás variables no tuvieron demasiado efecto a pesar de observarse algunas diferencias entre países.

Todos los países tienen una ley que establece la obligatoriedad de la vacunación, aunque en Chile, Costa Rica, Perú y Uruguay no aplica a todas las vacunas del calendario. Sin embargo, todos los referentes de los países expresaron dificultades en el cumplimiento de dicha obligatoriedad.

El SNRV tiene diferentes grados de implementación y alcance según el país. Al 2019, Uruguay contaba con el SNRV más desarrollado e implementado en su totalidad, Costa Rica no contemplaba el sistema público y privado, mientras que Brasil y Chile tenían pendiente conectarlo con los registros de nacidos vivos y con historia clínica electrónica (HCe) y, en Chile, faltaba también contemplar todas las vacunas de todas las edades y el sistema privado. Panamá solo incluía el sector público, y la conexión con la HCe y con el sistema de registros de nacidos vivos y defunciones estaba en proceso. Argentina y Paraguay contaban con un SNRV implementado de manera parcial en las provincias y la gestión de vacunaciones seguía siendo administrativa. Hasta el 2019, México no utilizaba un SNRV y solo contaba con una cartilla nacional de vacunación.

Por último, se evaluó el gasto teórico en vacunas en el 2019 por persona, que varió desde 2,78 dólares estadounidenses (US$) en Uruguay hasta US$5,21 en Panamá. Pero cuando se analizó según el producto bruto interno (PBI), el gasto varió desde 0,017% en Uruguay hasta 0,055% en Paraguay, con una tendencia inversamente proporcional al PBI per cápita de los países.

### Ranking general

**FIGURA 3.**
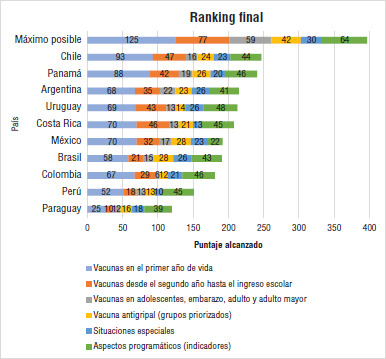
*Ranking* general de los programas de inmunizaciones de los países estudiados

En la figura 3 se observa el *ranking* general de los países con la suma de todas las variables. Está encabezado por Chile y Panamá, que se destacan a expensas de las vacunas y coberturas del primer y segundo año de vida. Luego se ubican Argentina, Uruguay y Costa Rica, que sobresalen en diferentes aspectos. Argentina lo hace en la vacunación de adolescentes, personas adultas y vacuna antigripal. Tanto Argentina como Uruguay obtienen un puntaje elevado en vacunación en situaciones especiales. Por otra parte, Uruguay y Costa Rica lo hicieron en la vacunación desde el segundo año de vida hasta el ingreso escolar.

México muestra un bajo puntaje en relación con la vacunación a partir del segundo año de vida hasta el ingreso escolar, con vacunas en adolescentes (tiene pendiente incorporar la dTpa y la vacuna contra el VPH en varones) y en los aspectos programáticos, a expensas principalmente de la falta de un SRNV.

*Fuente:* elaboración propia.

ESAVI, eventos adversos supuestamente atribuibles a la vacunación o inmunización; PBI, producto bruto interno; LATAM, América Latina; SNRV, sistema nominal de registro de vacunación.

Brasil muestra un puntaje reducido en casi todos los dominios, a excepción de la vacunación antigripal y en situaciones especiales. En Colombia se observa un puntaje bajo, especialmente en la vacunación desde el segundo año de vida y hasta las personas mayores, vinculado a la falta de vacunas inactivadas, acelulares y combinadas, la vacunación contra el VPH en varones, y dT y neumococo en personas adultas, así como también en la vacunación antigripal con menos grupos priorizados y coberturas más bajas que otros países.

Los países con puntaje más bajo en el *ranking* fueron Perú y Paraguay, que mostraron esquemas más atrasados de vacunación, coberturas bajas y vacíos de información.

### Discusión

El objetivo del presente trabajo fue construir un *ranking* de los PNI de América Latina que compare las diversas realidades, identifique los problemas y las metas no alcanzadas, y estimule a los países a la búsqueda de estrategias superadoras.

Si bien los PNI tienen una estructura similar en todos los países, en este trabajo correspondiente al año 2019, se observaron diferencias vinculadas al protagonismo que otorga cada ministerio de salud al programa de cada país.

Llama la atención la falta de información pública y de libre acceso por parte de los ministerios de muchos países. Esto podría corresponder, en ocasiones, a la ausencia del dato (p. ej., coberturas de vacunación en personas adultas) o, en otros casos, a la falta de difusión de estos. Esto último podría obedecer a que los países delegan dicha difusión a organismos como la OPS, que recopilan y publican los datos de forma periódica para toda la Región. Sin embargo, en el caso de los datos no solicitados por dichos organismos, se habrá perdido información relevante. Esto es particularmente evidente con vacunas de reciente incorporación o que no se encuentran incluidos en el PAI de la OPS como las vacunas contra la hepatitis A y la varicela y las vacunas para personas adultas (1).

*Fuente:* elaboración propia.

Este análisis resalta la problemática general de los PNI para alcanzar adecuadas coberturas que se evidencia en el bajo número de países con coberturas >95% para vacunas trazadoras y homogéneas a nivel municipal. En este sentido, contar en todos los países con un SNRV digital y de alcance global para todas las vacunas y subsectores de salud, contribuiría a disponer de la información en tiempo real y a generar estrategias para recuperar esquemas retrasados y alcanzar a la población relegada. Hace una década, se demostró en Australia el efecto que tiene un SNRV robusto sobre las coberturas de vacunación (40), y se reconfirmó durante la pandemia de COVID-19, cuando los países debieron vacunar prácticamente a toda su población en un tiempo récord. La disponibilidad de información relevante, confiable y oportuna fue clave para diseñar la campaña, para conocer la efectividad de las vacunas al momento que se implementaba la estrategia y para otorgar el certificado de vacunación (41).

Los países con mayor puntaje del *ranking*, como Chile, Panamá y Uruguay mostraron mayor uso de vacunas combinadas, inactivadas y acelulares, que, si bien son más costosas, simplifican la logística, el trabajo del personal de salud, la oportunidad de la vacunación y, en ocasiones, tienen un mejor perfil de seguridad. A su vez, estos países mostraron correlación con los PBI per cápita más altos de la Región, pero no con un mayor gasto en vacunas según el PBI. Esto último sí sucedió en Argentina, Paraguay y Perú, lo que pone en evidencia el mayor esfuerzo que deben realizar los países de menores ingresos para invertir en vacunas y alcanzar las mismas metas.

El análisis cuantitativo de las variables evaluadas en este estudio arroja como resultado un *ranking* general de los PNI de la Región con un patrón que se modifica al analizar de manera individual cada dominio. Este orden alternante evidencia las fortalezas y las debilidades de los programas en un mismo país y entre países en relación con diferentes aspectos evaluados (programáticos, situaciones especiales y vacunación antigripal) o etapas de la vida consideradas.

Existen diversos ejemplos de *rankings* de salud. El objetivo en común es la generación de datos útiles para la acción, para la búsqueda de respuestas y de estrategias que tengan un efecto positivo o, en el caso de los hospitales o universidades, de orientación para el usuario. Sin embargo, desde el punto de vista metodológico, los *rankings* ofrecen también dificultades, como la definición de las variables que se tienen en cuenta en la clasificación, la homogeneidad de la muestra a comparar, las fuentes de información disponibles y las ponderaciones, entre otras (42-44).

Este estudio tiene varias limitaciones. Por un lado, no se realizó una ponderación de las variables ni de las categorías y todas tuvieron un mismo valor. Por otra parte, el efecto que cada dominio y que las coberturas de vacunación tienen sobre el puntaje final se atribuyó a criterio de las autoras antes de comenzar a recopilar la información. En este sentido, el mayor valor atribuido a los dominios relacionados con vacunas de la infancia se pensó considerando que, hasta el ingreso escolar, se aplican las vacunas para prácticamente todas las enfermedades inmunoprevenibles y solo la vacuna contra el VPH se incorpora en la adolescencia, para proteger contra una nueva enfermedad. De la misma forma, el valor asignado a las variables vinculadas con coberturas vacunales en este ejercicio se fundamentó en la premisa de que lograr coberturas adecuadas es el pilar fundamental para garantizar el éxito de las estrategias aplicadas y las metas de control y eliminación de las enfermedades inmunoprevenibles. Asimismo, el puntaje asignado al SNRV también contribuye significativamente al puntaje total, y se elaboró de esta forma con el convencimiento de que un registro completo, informatizado y aplicado en la totalidad del territorio y de los diferentes subsistemas de salud es clave para conocer las coberturas reales de vacunación y las tasas de deserción, alcanzar a toda la población objetivo y dirigir las acciones de recupero de manera oportuna.

Por último, el gasto en vacunas analizado en nuestro trabajo fue teórico, con base en la población objetivo de las vacunas de calendario. En el estudio no se contempló la cobertura real de vacunación ni la proporción de la población alcanzada por otros subsistemas de salud que son proveedores de vacunas, por lo que su estimación se acercaría más a la realidad en países con tasas altas de cobertura y en aquellos donde la seguridad social o el sector privado tienen un papel mínimo en la vacunación.

## CONCLUSIONES

En conclusión, este primer análisis ha permitido hacer una comparación de los PNI de la Región y destacar sus similitudes y diferencias, y pone de manifiesto las fortalezas y debilidades de estos programas. Se resalta la necesidad en común de implementar y mejorar los sistemas de registro para, a su vez, mejorar las coberturas de vacunación, y en este sentido se insta a los países a aprovechar la oportunidad de mejora que la pandemia de COVID-19 generó en estos sistemas para el registro de vacunación contra el SARS-CoV-2 y extenderla a todas las vacunas del calendario.

La difusión de estos resultados pretende estimular a los países a trabajar en los desafíos pendientes y a acortar las brechas en relación con las metas aún no alcanzadas. La realización periódica de este análisis permitirá valorar y comparar la evolución y el posicionamiento de los programas en el tiempo.

## Declaración

Las opiniones expresadas en este manuscrito son únicamente responsabilidad de las autoras y no reflejan necesariamente los criterios ni la política de la *Revista Panamericana de Salud Pública* o de la OPS.
